# Testing Using the DCP Probe of a Subgrade Modeled from Difficult-to-Compact Sand in a Calibration Chamber

**DOI:** 10.3390/ma18153548

**Published:** 2025-07-29

**Authors:** Dariusz Tymosiak, Maria Jolanta Sulewska, Wanda Kokoszka, Marta Słowik, Ewa Błazik-Borowa, Dominik Ożóg, Monika Puchlik

**Affiliations:** 1 Faculty of Civil Engineering and Environmental Sciences, Bialystok University of Technology, 45E Wiejska Street, 15-351 Bialystok, Poland; m.sulewska@pb.edu.pl (M.J.S.); m.puchlik@pb.edu.pl (M.P.); 2 Faculty of Civil and Environmental Engineering and Architecture, Rzeszow University of Technology, 12 Powstancow Warszawy Street, 35-959 Rzeszow, Poland; wandak@prz.edu.pl (W.K.); d.ozog@prz.edu.pl (D.O.); 3 Faculty of Civil Engineering and Architecture, Lublin University of Technology, 38D Nadbystrzycka Street, 20-618 Lublin, Poland; m.slowik@pollub.pl (M.S.); e.blazik@pollub.pl (E.B.-B.)

**Keywords:** DCP test, degree of compaction *I_S_*, calibration chamber, difficult-to-compact sand

## Abstract

The aim of the article is to analyze the possibilities of using a lightweight dynamic cone probe DCP to determine the quality of compaction of surface layers of embankments (from 0.10 m to approx. 0.80 m below ground level). For this purpose, comparative tests of non-cohesive soil used for the construction of embankments were carried out using the DCP test and direct tests of the degree of compaction *I_S_* in a calibration chamber with the following dimensions: height 1.10 m and diameter 0.75 m. The subsoil was prepared from difficult-to-compact sand (*Sa*) with a uniformity coefficient of *C_U_* = 3.10 and curvature coefficient of *C_C_* = 0.99. The soil in the laboratory in the calibration chamber was compacted in layers using a vibratory plate compactor. A database for statistical analysis was obtained, *n* = 68 cases described by seven variables: *z*, *ρ*, *w*, *ρ_d_*, *I_S_*, *PI*, *N*_10_(*DCP*). It was found that the DCP probe can be used to assess the degree of compaction of embankments made of non-cohesive soil, using the developed relationship *I_S_* = *f*(*z*, *N*_10_(*DCP*)).

## 1. Introduction

Traditional direct methods of controlling soil compaction based on the degree of compaction *I_S_* are time-consuming and labor-intensive.

To determine the degree of compaction using classical methods, it is necessary to examine the bulk density *ρ* (e.g., using a cutting cylinder or a sand cone) of the soil in the embankment, then take a soil sample from the embankment and analyze the moisture content *w* in the laboratory (using the drying method [1]) and the maximum dry density *ρ_dmax_* (using the Proctor method [1,2]). These tests cause interruptions in the technological process of constructing the embankment.

Increasing requirements regarding the use of rapid control methods for the quality of embankment compaction have led to the search for new indirect methods of quickly assessing compaction parameters. Efforts are also being made to find correlations between compaction measurement results obtained using different methods. The aim of the comparative studies described in the article is to improve and simplify the process of ongoing quality control of compaction in the surface layers of embankments made of non-cohesive soils. A quick assessment of compaction parameters directly translates into the pace of earthwork operations.

There is considerable interest in simple indirect testing methods (field methods), which are characterized by ease and speed of measurement execution. There is also interest in the simplicity of operation and mobility of testing equipment, as well as the possibility of obtaining immediate results for compaction control. Such methods include dynamic probing techniques. Dynamic probing is also used to assess the bearing capacity and deformability of coarse-grained soil layers, to evaluate the geotechnical parameters of the tested layer, to assess the uniformity of the layer, to identify weak or strong soil layers, and to determine the top of the bedrock [3].

One of the methods for rapid quality control of compaction in surface soil layers is probing with the Dynamic Cone Penetrometer (DCP) in accordance with the International American Society for Testing and Materials Standard ASTM D6951-03:2003 [4]. The DCP has been used for many years, in many countries, especially in English-speaking countries [5,6,7,8,9]. In European countries, the DCP probe has been and still is rarely used.

The aim of this study was to investigate the possibility of using the DCP probe for compaction control of surface soil layers (from 0.00 m to approximately 0.80 m below ground level (b.g.l.)) in various types of embankments. In embankments, the degree of compaction *I_S_* is commonly used as a measure of soil compaction quality. *I_S_* is a symbol frequently used in the literature and in the Polish standard PN-S-02205:1998 [10]. In the European standard EN 16907-1:2018 [2], the degree of compaction is denoted by the symbol *D_Pr_* and explained as the achieved compaction level in %, related to the Proctor maximum dry density *ρ_dmax_.*

Additionally, the study examined whether there are correlations between penetration index *PI* or *N*_10_(*DCP*) values obtained using the DCP probe and reference *I_S_* values determined by direct methods. The established correlation could be applied in construction practice for the quick self-assessment of the compaction of layers of embankments made from non-cohesive soils.

In countries with a climate where periodic negative air temperatures occur, embankments are most often built from non-cohesive soils with an appropriate grain size. The soils for embankment construction should be non-frost-heaving and not frost susceptible. In countries where negative air temperatures do not occur, embankments can be built from cohesive soils—in this case, calibration of DCP sounding is also necessary for cohesive soils.

Moreover, the soil for the construction of embankments must be well compactable, so that the soil, after compaction, reaches the required values of the degree of compaction *I_S_*, specified for a given embankment depending on its intended use and load [2,10]. For example, according to PN-S-02205:1998 [10], the required *I_S_* values for road embankments range from 0.95 to 1.03, depending on the type of road and the depth b.g.l. of the tested fill layer. In sustainable management, it is beneficial to use local soils for the construction of embankments, even if they do not compact very well. This article presents studies using the DCP sounding of poorly compactable sand.

## 2. DCP Probe

### 2.1. DCP Probe Applications

The DCP (Dynamic Cone Penetrometer) was first mentioned in 1956. The pioneer of this device was Scala (in [11]) who proposed the device for testing unpaved road surfaces in Australia, with the following parameters: hammer mass *m* = 9 kg, hammer drop height *h* = 0.508 m (Kumar et al. [12] report a drop height *h* = 0.51 m), and a cone apex angle of 30° [13].

Ayers et al. [14] report that in the early 1980s, the cone-shaped DCP was not commonly used in the United States. During this period, other instruments were often used, such as the Clegg hammer [15] or the PANDA device [16].

The DCP device gained recognition from many researchers due to its rapid measurement capability, robustness, mobility, simple operation, low cost, and high efficiency.

The DCP device was standardized in the USA in 2003 [4]. The DCP is primarily used for testing the surface soil layers of embankments and subgrades composed of non-cohesive soils, to a maximum depth of 2.00 m below ground level (b.g.l.). It can also be used to assess railway embankment surfaces [17], highway and airport pavements, as well as unpaved surfaces [14]. The DCP device can be used to evaluate the quality of sand bedding compaction [18,19] and the subgrade of shallow foundations [20]. Using the DCP, it is possible to determine the number of pavement layers, the thickness of each layer, and the strength of a given layer—especially for rural roads [21]. Wu and Sargand [22] state that the DCP can be used to depths greater than 0.90 m b.g.l. Based on conducted studies, some researchers have determined that the DCP can be used to depths of up to 3.00 m b.g.l. [13] and even up to 10.00 m b.g.l. [23]. Wu and Sargand [22] suggest that DCP testing should be halted when the penetration depth exceeds 0.60 m and at least 10 consecutive blows result in a Penetration Index (*PI*) reading of less than 1 mm per blow. Salgado and Yoon [11] and Hashemi and Nikudel [23] do not recommend using the DCP for gravel. Webster et al. [24] emphasize that the DCP is not suitable for testing soils containing particles larger than 50.8 mm in diameter. Livneh [25] believes that the DCP test can be used to determine the California Bearing Ratio (*CBR*), based on established correlations, to a depth of 0.80 m b.g.l. Seyman [26] recommends using the DCP to identify weak spots in soil that is presumed to be uniformly compacted.

The DCP probe is used for indirect testing of many properties of cohesive and non-cohesive soils based on established correlations of the probe’s *PI* readings with other geotechnical parameters, such as *CBR*, shear strength *φ’*, resilient modulus *Mr*, relative density *D_r_*, modulus of subgrade reaction *E_S_*, moisture content *w*, dry unit weight *γ_d_* (in [19,27]). Example calibration equations taken from the literature are shown in Table 1.

The DCP probe was also used for comparative studies with other field instruments (in [19]): Falling Weight Deflectometer (FWD), Nuclear gauge, Soil Stiffness Gauge (SSG), Plate Load Test (PLT), and Clegg hammer.

There are a few reports in the literature about the use of the DCP probe for testing the degree of compaction index *I_S_*, defined according to Formula (2).

Juntasan et al. [28] describe calibration studies conducted in Northeast Thailand on four types of soils, classified according to AASHTO as: A-2-4 (silty or clayey gravel sand, with a number of tests *n* = 19), A-3 (fine sand, *n* = 10), A-4 (silty soil, *n* = 26), A-7-5 (clayey soil, *n* = 5). The soils were compacted using a small manual compactor in a circular concrete pit with a diameter of 0.5 m and a height of 0.3 m. The bulk density of the soil was directly measured using a sand cone, the optimum moisture content, and the maximum dry density using the standard Proctor method. A non-typical type of DCP probe with a cone diameter of 20 mm and a cone apex angle of 20° was calibrated, with a weight of 2.3 kg dropping from a height of 508 mm along a guiding rail. Two levels were marked on the lower part of the rod: the lower one at a height of 83 mm from the tip of the cone and the upper one at a height of 166 mm from the tip of the cone.

The number of blows of the weight *N* needed to drive the cone to a depth from the lower to the upper level, that is, to a depth of 83 mm (*N*_83mm_), was measured. Regression Equations (1)–(4) were developed for each soil group, in the form (1):

(1) $$y=a\cdot \ln\left(x\right)+b\;$$

where *y* is the degree of compaction, *x* is the number of DCP blows, and *a* and *b* are the parameters of the models.

Models with parameters in the ranges: a = 5.602–15.590, b = 58.478–89.878 were obtained. The proposed models, *I_S_* = *f*(*N*_83mm_) in each group of soils had high determination

Yang et al. [7] analyzed the effect of moisture and the compaction index on *PI*, *M_r_*, and *CBR*. The soil studied was high liquid limit clay (CH) with good gradation from central south China with *ρ_dmax_* = 1.76 t/m^3^ and *w_opt_* = 16.8%. Laboratory tests were conducted on samples measuring: diameter 152 mm, height 120 mm, compacted manually in a cylinder. Soil samples were tested at moisture levels ranging from *w* = 5–22%. The degree of compaction, denoted in the article by the symbol *K*, was 90%, 93%, and 96%. The tests were performed with a DCP probe with a cone of 20 mm in diameter and a vertex angle of 60°, with a weight of 8.0 kg dropped from a height of 575 mm.

The result of this study was the penetration ratio *PR* (mm/drop). The maximum penetration depth with the DCP probe was 120 mm. Based on 18 sets of test results, a regression equation for the degree of compaction *K* was developed. The tests were repeated in the field on an embankment from which samples were taken for laboratory testing, at 62 measurement points, with a penetration depth using the DCP probe of up to 300 mm p.p.t. The bulk density *ρ* tests were conducted using the sand cone method. The regression equations for *K* from laboratory and field studies are presented in Table 1. It was found that the results of the *PR* probing depend on the soil moisture and its degree of compaction *K*. The two-factor linear regression equations *K* = *f*(*w*, *PR*) have correlation coefficients *R* > 0.80.

The research by Belicanta et al. [29] aimed to develop an equation for interpreting the results of soil tests using the DCP probe. The DCP probe had a cone with a diameter of 20 mm and a vertex angle of 60°, with a weight of 8.0 kg falling from a height of 575 mm. The value of *PI* (cm/blow) was calculated as the average penetration value from 10 consecutive blows of the weight. The tests were conducted in the field on the soil in the city of Bandairantes, Parana, Brazil: lateritic silty clay, evolved from basaltic rock. In the field, *PI* (cm/blow), the unit weight of the soil *γ* (kN/m^3^) was measured using the sand cone method, and the moisture in (%) was determined by drying. Standard Proctor tests were conducted on the collected samples, resulting in average values of *γ_dmax_* = 16.3 kN/m^3^, *w_opt_* = 23.5%.

It has been found that the *PI* value is inversely proportional to the density index and is dependent on soil moisture. For the studied soil, an equation was developed for estimating the degree of compaction, denoted by the symbol *DC*, as a function of *PI* and *w*, shown in Table 1. Data analysis showed that for the studied soil, the calibration equation has great potential for application in construction, with a recommendation that the tested layer should not be thicker than 200 mm.

From the above literature review, it is evident that the DCP probe has mostly been calibrated in countries with warm climates, for practical use in monitoring the compaction of specific embankments. Laboratory studies were conducted on small-sized samples. The studies presented were mostly carried out on cohesive soils. In the equations by Yang et al. [7] and Belicanta et al. [29], the variable moisture was included as an independent variable, which is considered a redundant variable here, as it is used to calculate the dependent variable, the degree of compaction, according to Equations (2) and (3). The overall conclusion from the presented studies is that the value of the degree of compaction depends on the soil compaction, as indicated by the value of the penetration index (*PI*). The results of the presented studies are difficult to generalize to other than the studied groups of soils; therefore, the authors of the cited articles advocate calibrating the DCP probe each time for specific soils.

This article presents studies on non-cohesive soil—poorly graded sand that is difficult to compact. The studies were conducted in a calibration chamber with a diameter of 750 mm, simulating field conditions regarding soil volume. Calibration tests were performed at depths from 0.00 to approximately 800 mm below ground level.

The next soils we will study will be non-cohesive soils with coarser grading, well-graded, and easily compactable. Then it will be possible to determine whether the calibration equations can be generalized for all studied non-cohesive soils or for specific groups of soils.

### 2.2. Interpretation of DCP Soil Testing Results

The DCP device is one of the indirect methods of soil testing—it is calibrated for a specific purpose, i.e., empirical equations are developed to relate the readings obtained from the test with other sought-after geotechnical parameters.

The result of the soil test using the conical DCP device is the *PI* (Penetration Index), or *DPI* (Dynamic Penetration Index), or *DCPI* (Dynamic Cone Penetration Index), measured in mm per blow [4,27,30].

Regression equations for the *PI* with the California Bearing Ratio (*CBR*) or Relative Density *D_r_* have been most commonly developed (Table 1). Research is still ongoing regarding the use of the DCP device for evaluating various geotechnical parameters.

**Table 1:** Examples of correlations obtained from DCP probe results.

Authors (Year)	Formula	Scope of Application	Coefficient of Determination
Webster et al. (1992) [24]	*CBR* = 292/*DCP*^1.12^	cohesive soils and non-cohesive soils	Not specified
Zohrabi and Scott (2003) [31]	*CBR* = 240 *DCP*^−0.97^	cohesive soils and non-cohesive soils	*R*^2^ = 0.89
	*CBR* = 243 [*DCP*/(1 + *w*)]^−1.01^		*R*^2^ = 0.90
George et al. (2009) [5]	*CBR* = 47.32 (*DCPI*)^−0.7852^	lateritic soils	*R*^2^ = 0.82
Jordão et al. (2012) [32]	*CBR* = 980.55 *DCP*^−1.257^	silty clay	*R*^2^ = 0.86
Monteiro et al. (2016) [33]	*CBR* = 143.13 (*DCP*)^−0.879^	silty or clayey gravel and sand	*R*^2^ = 0.89
	*CBR* = 43.91 (*DCP*)^−0.547^	clayey soils	*R*^2^ = 0.71
	*CBR* = 58.154 (*DCP*)^−0.397^	stone fragments, gravel, and sand	*R*^2^ = 1.00
Hamid (2017) [18]	*D_r_* = 230.55/(DCPI)^0.417^	silty sand; *C_U_* = 1.98; (+1% silt)	*R*^2^ = 0.98
	*D_r_* = 231/(*DCPI*)^0.419^	silty sand; *C_U_* = 1.98; (+4% silt)	*R*^2^ = 0.98
	*D_r_* = 214/(*DCPI*)^0.405^	silty sand; *C_U_* = 1.98; (+8% silt)	*R*^2^ = 0.98
Wilches et al. (2018) [34]	*CBR* = 112.03/(*DCP*)^0.803^	fine-grained soils, clay	*R*^2^ > 0.80
MacRobert et al. (2019) [9]	*D_r_* = −50 log*DPI* + 148	sandy soils; *C_U_* = 1.2–9.4	*RE* = ±11%
	*D_r_* = −52 log(*DPI · D*_50_)^0.3^ + 150		*RE* = ±9%
Juntasan et al. (2015) [28]	*I_S_* = 5.785 ln(*N*_83mm_) + 86.654	silty or clayey gravel sand; *ρ_dmax_* = 1.821 t/m^3^; *n* = 19	*R*^2^ > 0.96
	*I_S_* = 8.751 ln(*N*_83mm_) + 73.902	fine sand; *ρ_dmax_* = 1.867 t/m^3^; *n* = 10	*R*^2^ > 0.96
Yang et al. (2015) [7]	*K* = 131.57 − 1.05*w* − 5.00*PR* for *w* ≤ *w_opt_* *K* = 46.76 − 3.18*w* − 1.79*PR* for *w* ≥ *w_opt_*	clay (CH); *C_U_* = 5.59 Laboratory tests	*R*^2^ = 0.76 *R*^2^ = 0.67
	*K* = 134.57 − 1.08*w* − 5.39*PR* for *w* ≤ *w_opt_*; *n* = 18 *K* = 49.36 − 3.47*w* − 1.92*PR* for *w* ≥ *w_opt_*; *n* = 44	clay (CH); *C_U_* = 5.59 Field tests	*R*^2^ = 0.81 *R*^2^ = 0.80
Belicanta et al. (2016) [29]	$DC=\frac{100\cdot \gamma_{d}}{16.3}=\frac{210}{PI}\cdot e^{9.5\left(\frac{w-26.5}{26.5}\right)}$; *PI* (cm/blow)	lateric silty clay	No data

*CBR*—California Bearing Ratio; *DCP* or *DPI* or *DCPI* or *PR* or *PI*—the penetration of the cone tip of the DCP device in millimeters caused by one hammer blow; *w*—soil moisture content; *w_opt_*—optimum moisture content; *D_r_*—relative density of the soil; *D*_50_—grain diameter in mm, for which 50% of the soil particles are smaller; *C_U_*—uniformity coefficient (gradation); *Is* or *K* or *DC*—degree of compaction; *N*_83mm_—number of DCP blows required for 83 mm penetration; *ρ_dmax_*—maximum dry density; e ≈ 2.718—Euler’s number; $\gamma_{d}$—dry unit weight; *R*^2^—coefficient of determination; *RE*—relative error; *n*—number of studies.

### 2.3. Description of the DCP Device

**Figure 1:**
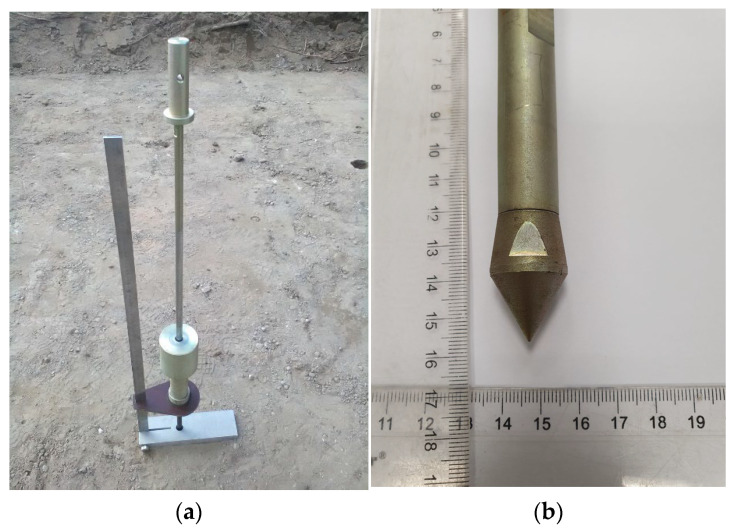
DCP device according to ASTM D6951-03:2003 [4]: (**a**) view of the DCP device; (**b**) conical tip of the DCP (photo by D. Tymosiak).

The DCP device and its conical tip are shown in Figure 1, and the technical specifications of the device are presented in Table 2.

## 3. Description of the Tests Performed

### 3.1. Description of the Tested Soil

**Figure 2:**
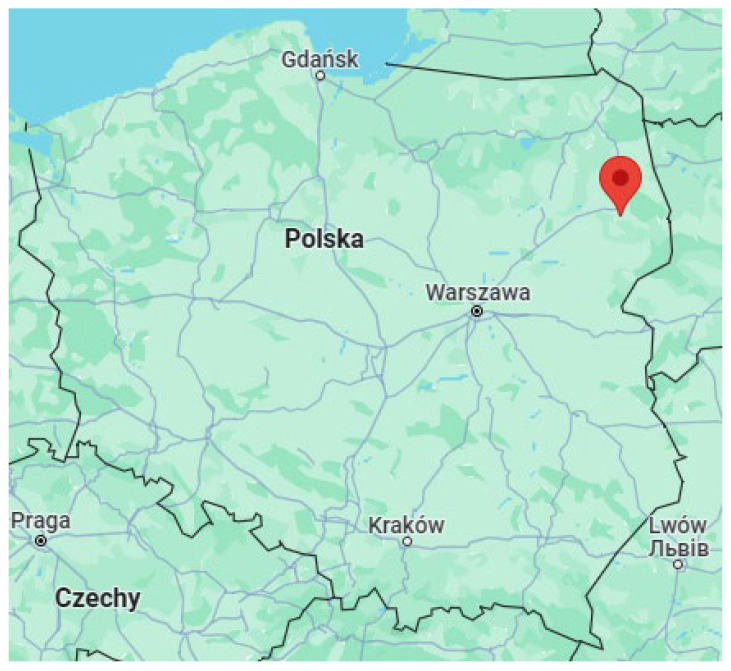
Location of the site where soil was taken for testing near the city of Bialystok (marked with a red symbol), Poland. Map data ©2025 Google (www.google.com/maps, accessed on 30 April 2025).

The soil substrates for testing in the calibration chamber [35] were prepared from non-cohesive soil of post-glacial origin taken near the city of Bialystok, Poland (Figure 2).

**Figure 3:**
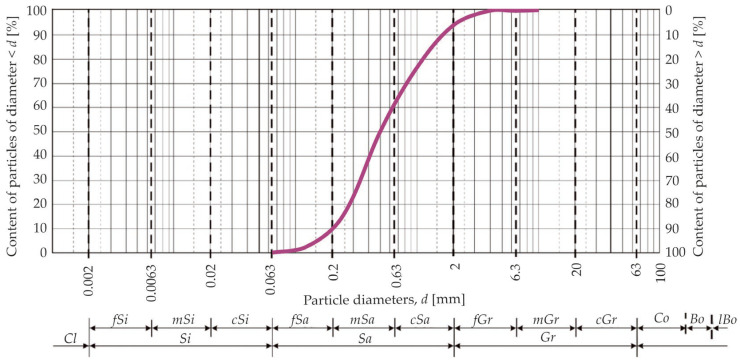
Sand (*Sa*) grain size curve [35].

The name of the studied soil was read from the triangle in the European standard EN ISO 14688-2:2004 [36]: sand (*Sa*). The grain size distribution of the soil was determined using the sieve analysis method in accordance with CEN ISO/TS 17892-4:2004 [37]. The grain size distribution curve of the tested sand is shown in Figure 3. Grain size and the geotechnical parameters of the soil are presented in Table 3. Based on the values of the uniformity coefficient (*C_U_*) and the coefficient of curvature (*C_C_*), it was determined that the tested sand was poorly graded, and therefore difficult-to-compact, according to EN ISO 14688-2:2004 [36].

**Figure 4:**
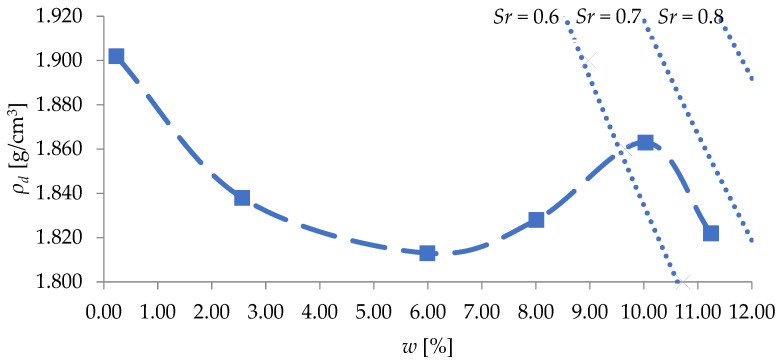
Compaction curve of sand (*Sa*) obtained using the standard Proctor method [39], where *ρ_d_* is the dry density; *w* is the moisture content; *Sr* is the degree of saturation (i.e., the proportion of pore space filled with water), indicated by the dotted line.

Compaction parameters of the tested soil: the maximum dry density *ρ_dmax_* and the optimum moisture content *w_opt_* were determined using the standard Proctor method according to the Polish standard PN-B-04481:1988 [1], which corresponds to A+A method as per EN 13286-2:2010 [38], using a mechanical Proctor apparatus. The compaction curve of the tested sand is shown in Figure 4.

### 3.2. Calibration Chamber

**Figure 5:**
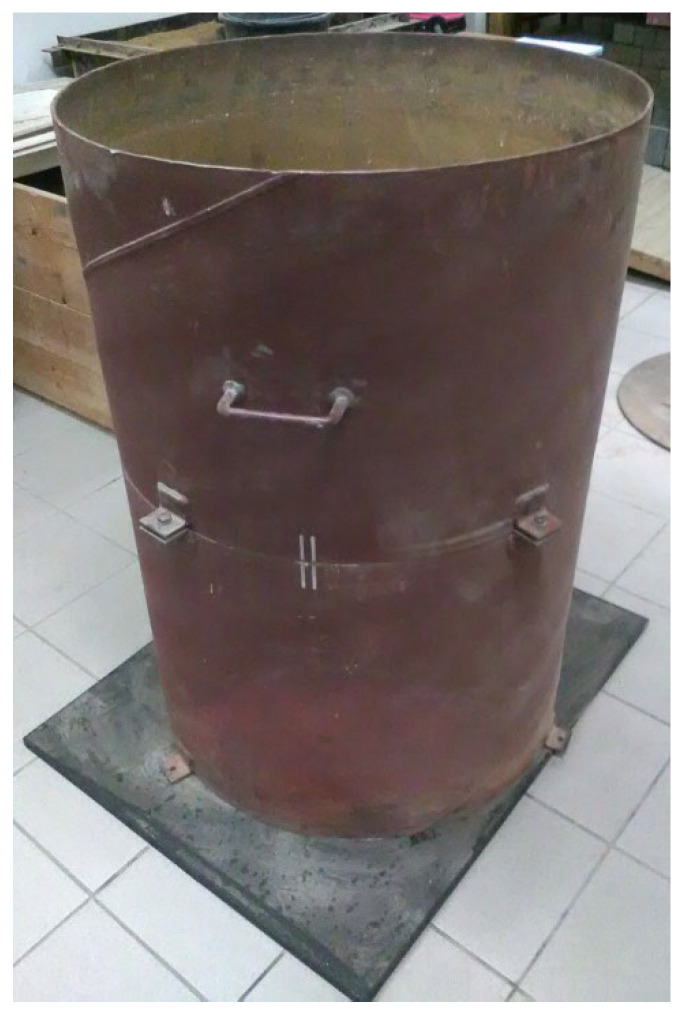
Calibration chamber (photo by D. Tymosiak).

The test beds were prepared in a steel calibration chamber with dimensions: height 1.10 m, diameter 0.75 m, and wall thickness 5.8 mm. The calibration chamber was placed on a rigid floor, standing on a rubber pad (Figure 5).

### 3.3. Substrate Modeling in a Calibration Chamber

**Figure 6:**
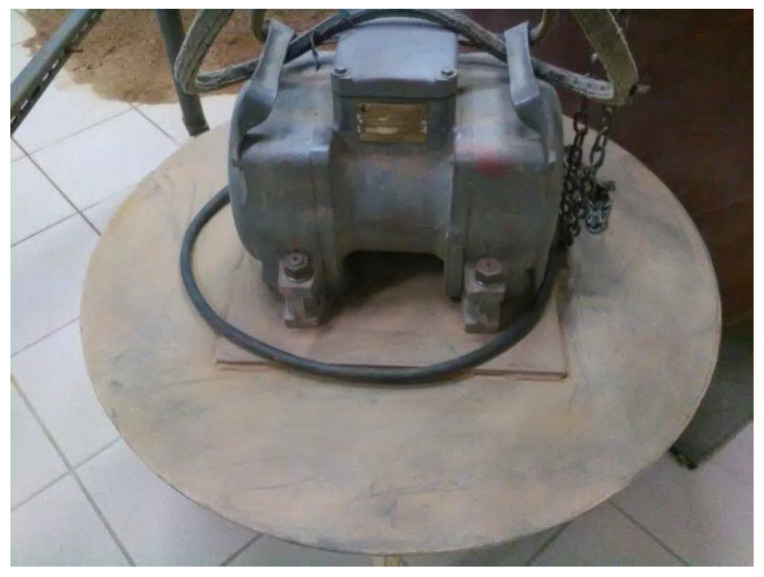
Vibratory plate compactor EWC 22A (photo by D. Tymosiak).

The soil in the calibration chamber was compacted in layers approximately 0.25 m thick using an EWC 22A vibratory plate compactor (Figure 6).

The technical parameters of the EWC 22A vibratory plate compactor were as follows: frequency 50 Hz, total weight 73 kg, compaction plate diameter 720 mm, and plate thickness 6.5 mm.

A total of eight model test beds were prepared. To vary the compaction parameters, the soil was compacted in 3 or 4 layers, with post-compaction thicknesses ranging from 0.14 m to 0.29 m. To achieve different levels of compaction, each layer was compacted for different durations: from 1 min to 20 min. The total thickness of the compacted model bed ranged from 0.75 m to 0.81 m. The sand was compacted at various moisture contents.

### 3.4. Measures of Density Tested

For artificially compacted soils (both cohesive and non-cohesive), the degree of compaction *I_S_* is the direct measure used to assess the quality of compaction in embankment soils during earthworks. The directly measured values of *I_S_* were calculated using the following Formula (2):

(2) $$I_{S}=\frac{\rho_{d}}{\rho_{dmax}}=\frac{\gamma_{d}}{\gamma_{dmax}}$$

where *ρ_d_* or $\gamma_{d}$ is the dry density or dry unit weight of the soil in the calibration chamber [g/cm^3^] or [kN/cm^3^]; *ρ_dmax_* or $\gamma_{dmax}$ is the maximum dry density or max dry unit weight [g/cm^3^] or [kN/cm^3^] at the optimum moisture content *w_opt_*, determined in the laboratory using the standard Proctor method according to [1,38].

The dry density of the soil *ρ_d_* was calculated according to Formula (3):

(3) $$\rho_{d}=\frac{100\cdot \rho }{100+w}$$

where *ρ* is the bulk density of the soil, tested with two measuring cylinders with volumes of 205.17 cm^3^ and 43.96 cm^3^ according to CEN ISO/TS 17892-2:2004 [40] and calculated as the average value of the two cylinders; *w* is the moisture content of the soil tested by the drying method according to CEN ISO/TS 17892-1:2004 [41] and calculated as the average value of the moisture content of the two samples.

### 3.5. Performing Tests

**Figure 7:**
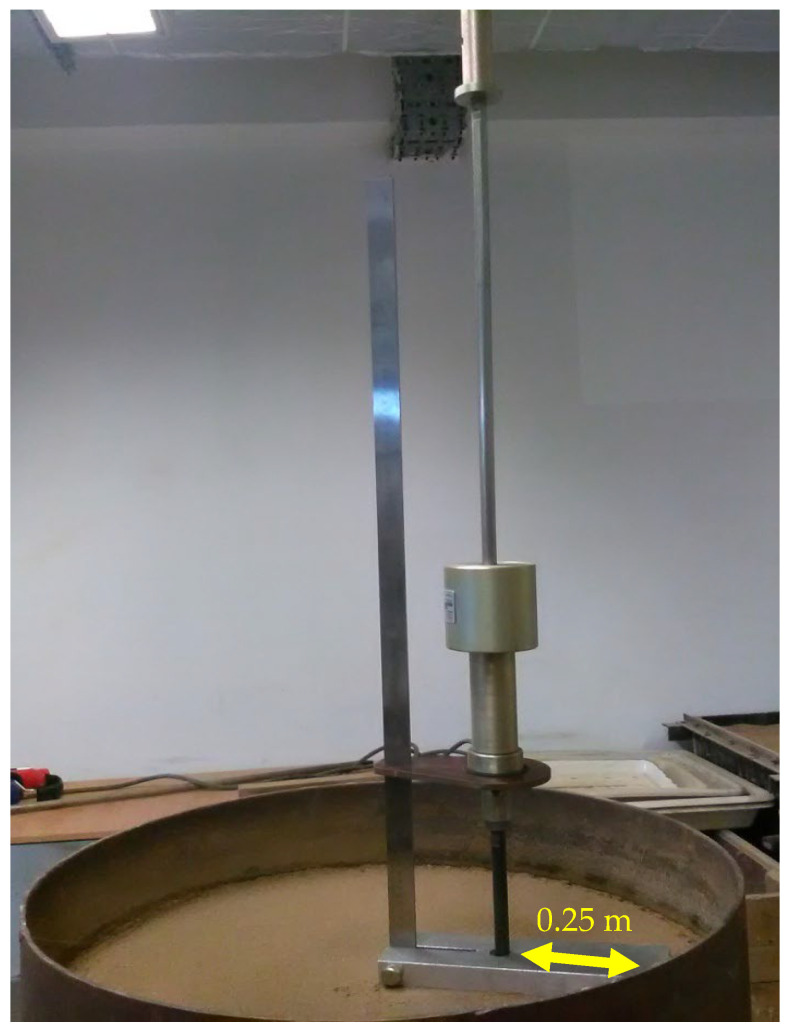
Examination of the modeled substrate with the DCP probe (photo by D. Tymosiak).

The testing of the model subgrade in the calibration chamber was carried out in the following sequence. The DCP probe was positioned vertically on the leveled surface of the subgrade prepared in the calibration chamber, at a minimum distance of 0.25 m from the chamber walls, in accordance with the recommendations of Nguyen and Mohajerani [6] (Figure 7). The hammer was lifted along the guide to a height of 0.575 m and then released freely. After each blow of the hammer, the penetration depth of the cone (*PI* in mm) was read from the attached vertical ruler. These steps were repeated until the cone penetrated through the full thickness of the prepared subgrade.

The number of blows (*N*_10_) required for 10 cm of DCP probe penetration was calculated and recorded as *N*_10_(*DCP*), similarly to the light dynamic probing test (DPL) according to EN ISO 22476-2:2005 [42]. Based on consistent successive *N*_10_(*DCP*) values, the thicknesses of subgrade layers with similar compaction levels were determined. Next, the soil in the calibration chamber was excavated layer by layer, as defined above, to a specified depth *z*, at which point measuring cylinders were pressed into the soil to determine the bulk density *ρ*, and samples were collected for determining the moisture content *w*. Then, the dry density of the soil *ρ_d_* was calculated using Equation (3), and the degree of compaction *I_S_* was calculated using Equation (2) for each layer of the model subgrade.

## 4. Results

### 4.1. Summary of Research Results

**Figure 8:**
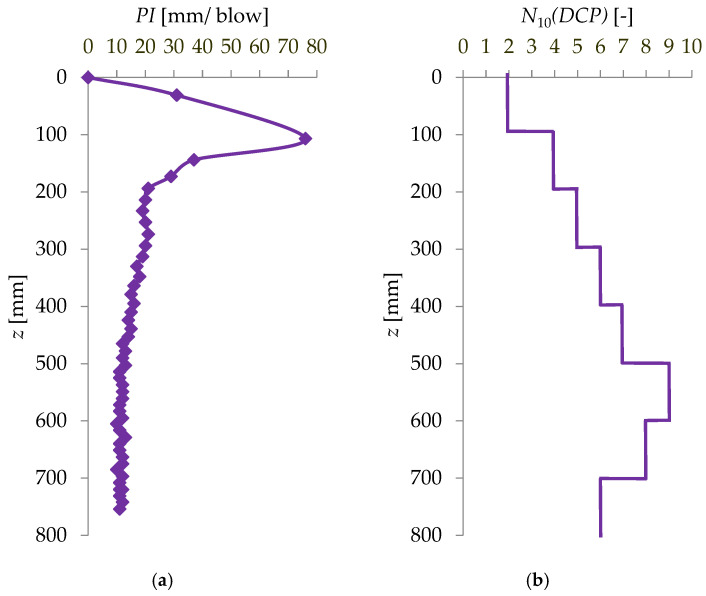
Sample DCP probing charts for subgrade No. 1 [35]: (**a**) relationship between *PI* and penetration depth *z*, (**b**) relationship between the number of blows *N*_10_(*DCP*) and penetration depth *z*.

Example graphs of DCP sounding results are shown in Figure 8.

A database named Dataset1 was created, consisting of *n*_1_ = 68 cases, described by seven variables: *z*, *ρ*, *w*, *ρ_d_*, *I_S_*, *PI*, and *N*_10_(*DCP*).

Next, a database named Dataset2 was created, consisting of *n*_2_ = 42 cases, described by the same seven variables: *z*, *ρ*, *w*, *ρ_d_*, *I_S_*, *PI*, and *N*_10_(*DCP*), with the exception that the test results for *z* from 0.0 m to *z* = 0.20 m b.g.l. were removed in order to eliminate the maximum settlement values observed at depths from 0.00 to approximately 0.20 m b.g.l.

The range of measurement results for the eight subgrade models in Dataset1 and Dataset2 is shown in Table 4.

It can be observed that the coefficient of variation for *PI* in Dataset1 is *V*_1_ = 58.5%, while for *N*_10_(*DCP*), it accounts for *V*_1_ = 69.0%. After eliminating the initial settlement increase, in Dataset2, the coefficients of variation of these parameters decreased to *V*_2_ = 40.5% and *V*_2_ = 40.4%, respectively. The coefficients of variation for the geotechnical parameters *ρ_d_* and *I_S_* were low, about 2.0% in both datasets, which may indicate that the compaction in the surface layer at *z* = 0.0–0.2 m b.g.l. is similar to the compaction in deeper layers.

### 4.2. Statistical Analysis of Test Results

The statistical analysis of the research results contained in the databases according to Table 4 was performed using the computer program STATISTICA version 13.3 [43,44]. Two issues were analyzed:

(1) Which geotechnical parameters are dependent on *PI*?
(2) Can relationships be developed for the degree of compaction *I_S_* based on the results obtained from DCP tests?

A simple linear regression model was considered according to Equation (4):

(4) y=a+by±ε

If the parameter *a* significantly differs from 0, a systematic error in the method occurs. When the slope coefficient *b* significantly differs from 1, a random error occurs. The correlation coefficient *r* between variables *y* and *x* should significantly differ from 0. The significance of the correlation coefficient *r* also depends on the number of cases *n* in the dataset. For a sample size of *n*_1_ = 68, a statistically significant correlation coefficient should be *r* ≥ 0.24, and for *n*_2_ = 42, it should be *r* ≥ 0.31 [45].

Additionally, a multiple linear regression model was considered according to Equation (5):

(5) $$y=a_{0}+a_{1}x_{1}+\cdots +a_{n}x_{n}\pm \varepsilon$$

In Equations (4) and (5), *y* are dependent variables; *x*, *x*_1_, *…*, *x_n_* are independent variables; *a* and *a*_0_ are the intercepts; *b*, *a*_1_, *…*, *a_n_* are the coefficients of the equations; *ε* is the standard error of estimation [44]. In addition, nonlinear models were considered [44].

The error measures of the models are: the correlation coefficient *r*, the coefficient of determination *R*^2^ = *r*^2^, and the relative error *RE* expressed by Equation (6):

(6) $${RE}_{i}=\left|\frac{y_{i}\;-\;{\hat{y}}_{i}}{y_{i}}\right|100\%$$

in which $y_{i}$ are the known values of the dependent variable from the studies; ${\hat{y}}_{i}$ are the values of the dependent variable predicted by the model.

Further statistical analyses were performed on the data from Dataset1 and Dataset2, and statistical inference was carried out at the adopted test probability level (*p* = 0.05) [43,44,46].

The compatibility of the distributions of the variables *I_S_*, *PI*, and *N*_10_(*DCP*) with the normal distribution was verified using the Kolmogorov–Smirnov (K-S) test. It was found that the distributions of the studied variables in both datasets correspond to the normal distribution (*p* > 0.20 and *p* < 0.10 for Dataset1, and *p* > 0.20 for Dataset2).

Pearson linear correlation coefficients *r* were calculated [43]. The linear correlation matrices for Dataset1 and Dataset2 are presented in Table 5, where statistically significant correlation coefficients *r* are marked in bold font with colorful backgrounds, indicating the strength of correlation between the variables.

It can be observed that in Dataset1, the values of *PI* and *N*_10_(*DCP*) mainly depend on the depth of the test *z* (with *r* = −0.751 and *r* = 0.809), and the degree of compaction *I_S_* (with *r* = −0.260 and *r* = 0.440). Of course, the value of the compaction index *I_S_* is strongly dependent on the bulk density *ρ* and the soil moisture *w*, based on which the dry density *ρ_d_* is calculated using Formula (3).

In Dataset2, the values of *PI* and *N*_10_(*DCP*) depend on the degree of compaction *I_S_* (with *r* = −0.512 and *r* = −0.512), and to a somewhat lesser extent, on the depth of the test *z* (with *r* = −0.409 and *r* = −0.409).

The developed statistically significant simple and multiple linear and nonlinear regression equations according to Formulas (7)–(17) for Dataset1 and Dataset2 are presented in Table 6.

Based on Table 6, it can be seen that the multiple regression models for the dependent variables *PI* and *N*_10_(*DCP*), expressed by Equations (11) and (12) in Dataset1, have the highest coefficients of determination *R*^2^ = 0.613 and *R*^2^ = 0.806, respectively. This means that these equations explain 61.3% and 80.6% of the variability in the dependent variables. The *R*^2^ values in Dataset1 are higher than those in Dataset2, so it can be concluded that the DCP test results can be interpreted from the surface of the terrain, and the results obtained in the 0.00–0.20 m b.g.l. range should not be discarded. Moreover, in Dataset1 the variables have a wider range of variability.

Therefore, the possibility of developing calibration equations in Dataset1 for the DCP probe was analyzed. Based on these equations, the value of the degree of compaction *I_S_* of the tested sand could be determined using the measured values of *PI* or *N*_10_(*DCP*) and the depth of testing *z*.

The models *Is* = *f*(*PI*) and *Is* = *f*(*z*, *PI*) were rejected due to very low *R*^2^ values, 0.068–0.128 and 0.085–0.222, respectively, or they are statistically insignificant.

Next, the model *Is* = *f*(*z*, *N*_10_(*DCP*)) was analyzed. Since the values of *N*_10_(*DCP*) are correlated with the depth of the test *z* (as shown in Table 5), multicollinearity of the independent variables was checked using the calculated variance inflation factor (*VIF*) and tolerance analysis, as well as partial and semi-partial correlation [44,47]. Since the “pure” contribution of variable *z* in explaining the dependent variable *I_S_* was slightly smaller than that of variable *N*_10_(*DCP*), the *VIF* for variable *z* was calculated as *VIF* = 3.20. According to Daoud [48], 1 < *VIF* ≤ 5 means moderate correlation. Therefore, the selected best model was adopted in the form of Equation (18):

*I_S_* = 0.927 – 0.069*z* + 0.005*N*_10_(*DCP*) ± 0.013 with *R*^2^ = 0.412 (18)

**Figure 9:**
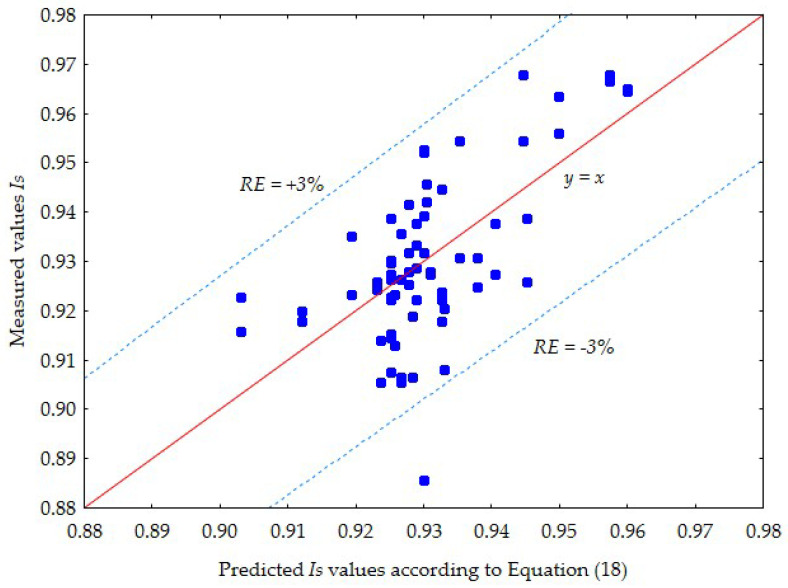
A comparison of the predicted *Is* values according to Equation (18) and the measured values *I_S_*.

The adopted model is statistically significant (at *p* ˂ 0.00000). All words of the selected model are statistically significant (at *p* = 0.00000). Analysis of the residuals showed that the residuals are uncorrelated and have a normal distribution [44]. Although it explains only 41.2% of the variability of the dependent variable, it predicts the degree of compaction with a relatively small relative error of approximately *RE* = ±3%, which is sufficient for practical purposes. A comparison of the predicted *Is* values according to Equation (18) and the measured values is shown in Figure 9.

## 5. Conclusions

In the study, the DCP probe was calibrated for its application for the indirect assessment of the degree of compaction *I_S_* in surface embankment layers. The reference values were obtained from direct degree of compaction tests. The tested subgrades were modeled from sand (*Sa*) in a calibration chamber to eliminate potential heterogeneities in grain size distribution, moisture content, and compaction. Based on the research results, the following conclusions can be drawn:

- The results of DCP testing can be interpreted from the ground surface.
- The DCP test results (*PI* and *N*_10_(*DCP*)) of sand mainly depend on the test depth *z* and the degree of compaction *I_S_*.
- A statistically significant calibration equation for the DCP probe was developed, which allows determining the degree of compaction *I_S_* of the tested sand (*Sa*) based on the measured values of *N*_10_(*DCP*) and test depth *z*, with a relative error of *RE* = ±3%: *I_S_* = 0.927 − 0.069*z* + 0.005*N_10_*(*DCP*) ± 0.013.
- Based on the conducted research and analyses, it is concluded that the DCP probe can be used to assess the compaction index of surface layers of embankments made of poorly graded non-cohesive soil (sand (*Sa*)), using the developed regression equation *Is* = *f*(*z*, *N*_10_(*DCP*)).
- The DCP probe has great application possibilities for studying the degree of compaction of soils. In this article, the DCP probe was calibrated on one soil type: poorly graded sand. The probe should be calibrated on other cohesionless soils with different grain size distributions.
- Studies will be undertaken on several cohesionless soils with coarser grain sizes, with medium, well, and gap graded.
- Calibration equations for individual soils will be developed. The possibility of generalizing the calibration equations for all cohesionless soils or groups of cohesionless soils will be examined.
- The developed calibration equations will be verified based on field studies on road embankments.

## Figures and Tables

**Table 2:** Technical parameters of the DCP device according to ASTM D6951-03:2003 [4].

Parameter	Value of Parameter
Hammer mass *m* [kg]	8 ± 0.01
Hammer drop height *h* [mm]	575 ± 10
Apex angle of the cone [°]	60 ± 1
Diameter of the base of the conical tip *d* [mm]	20.0 ± 2.5
Mass of the rod [kg/m]	1.55
Outer diameter of the rod [mm]	15.8
Impact energy of the hammer per unit area [kJ/m^2^]	143.7
Test result *PI* [mm/1 blow]	*PI* (*Penetration Index*)

**Table 3:** Geotechnical parameters of the tested sand (*Sa*) [35].

Geotechnical Parameter	Symbol	Value
Fraction content:	*fgr*; *csa*; *mSa*; *fsa*	5%; 35%; 50%; 10%
Grain size distribution: *D*_10_, *D*_30_, *D*_60_ (grain diameters for which 10%, 30%, and 60% of the soil sample, respectively, are finer)	*D*_10_	0.200 mm
	*D*_30_	0.350 mm
	*D*_60_	0.620 mm
Uniformity coefficient	*C_U_* = *D*_60_/*D*_10_	3.10
Curvature coefficient	*C_C_* = (*D*_30_)^2^*/D*_10_*·D*_60_	0.988
Maximum dry density of the soil	*ρ_dmax_*	1.863 g/cm^3^
Optimum moisture content	*w_opt_*	10.03%
Bulk density of the soil	*ρ*	1.717–1.903 g/cm^3^
Moisture content of the soil	*w*	3.40–5.81%
Dry density of the soil	*ρ_d_*	1.649–1.803 g/cm^3^

**Table 4:** The range of values of the tested geotechnical parameters.

Parameter	Dataset1			Dataset2		
	Values Range	Standard Deviation *S*_1_	Coefficient of Variation *V*_1_ [%]	Values Range	Standard Deviation *S*_2_	Coefficient of Variation *V*_2_ [%]
*z* [m]	0.02–0.70	0.23	68.3	0.21–0.70	0.15	30.3
*ρ* [g/cm^3^]	1.732–1.903	0.037	2.1	1.756–1.903	0.042	2.3
*w* [%]	3.4–5.8	0.6	12.6	3.8–5.8	0.6	12.1
*ρ_d_* [g/cm^3^]	1.650–1.803	0.032	1.9	1.687–1.803	0.034	2.0
*I_S_* [-]	0.886–0.968	0.017	1.9	0.906–0.968	0.019	2.0
*PI* [mm/1 blow]	6.0–57.0	12.9	58.5	6.0–30.0	5.7	40.5
*N*_10_(*DCP*) [blow number/10 cm]	1.0–17.0	4.0	69.0	4.0–17.0	3.4	40.4

**Designation: **The gray color marks the Dataset2.

**Table 5:** Pearson linear correlation *r* coefficient matrix.

Dataset1							
Variable	*z*	*ρ*	*w*	*ρ_d_*	*I_S_*	*PI*	*N*_10_(*DCP*)
*z*	1.000						
*ρ*	0.146	1.000					
*w*	**0.239**	**0.498**	1.000				
*ρ_d_*	0.100	**0.962**	**0.253**	1.000			
*I_S_*	0.100	**0.962**	**0.253**	**1.000**	1.000		
*PI*	**−0.751**	−0.228	0.012	**−0.260**	**−0.260**	1.000	
*N*_10_(*DCP*)	**0.809**	**0.463**	**0.286**	**0.440**	**0.440**	**−0.776**	1.000
**Dataset2**							
**Variable**	***z***	***ρ***	***w***	***ρ_d_***	***I_S_***	***PI***	***N*_10_(*DCP*)**
*z*	1.000						
*ρ*	0.025	1.000					
*w*	0.240	**0.603**	1.000				
*ρ_d_*	−0.026	**0.975**	**0.416**	1.000			
*I_S_*	−0.026	**0.975**	**0.416**	**1.000**	1.000		
*PI*	**−0.409**	**−0.451**	−0.022	**−0.512**	**−0.512**	1.000	
*N*_10_(*DCP*)	**0.484**	**0.632**	**0.369**	**0.625**	**0.625**	**−0.831**	1.000

**Designations:** Pearson’s r linear correlation coefficient. 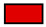 0.99–0.60; 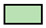 (−0.21)–(−0.60); 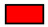 0.59–0.20; 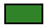 (−0.61)–(−0.99); 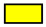 0.19–(−0.20).

**Table 6:** Regression equations in Dataset1 and in Dataset2.

Dataset1			
Function	Regression Equation	*R* ^2^	Formula No.
*PI* = *f*(*z*)	*PI* = 46.9 − 108.2*z* + 82.2*z*^2^ ± 8.3	0.600	(7)
*PI* = *f*(*I_S_*)	*PI* = 203.7 − 195.1*I_S_* ± 12.6	0.068	(8)
*N*_10_(*DCP*) = *f*(*z*)	*N*_10_(*DCP*) = 11.9 + 11.0log*z* ± 2.3	0.681	(9)
*N*_10_(*DCP*) = *f*(*I_S_*)	*N*_10_(*DCP*) = −5368.3 + 5392.6*I_S_* – 11,369.0log*I_S_* ± 3.6	0.273	(10)
***PI* = *f*** **(*z*, *I_S_*)**	***PI* = −31.2log*z* − 293.3log*I_S_* ± 8.2** (the intercept is statistically insignificant)	**0.613**	**(11)**
***N*** **_10_** **(*DCP*) = *f*(*z*, *I_S_*)**	***N*** **_10_** **(*DCP*) = 17.3 + 10.5log*z* + 182.6log*I_S_* ± 1.8**	**0.806**	**(12)**
**Dataset2**			
**Function**	**Regression equation**	***R*** **^2^**	**Formula No.**
*PI* = *f*(*I_S_*)	*PI* = 86.6 − 83.4*I_S_*^2^ ± 5.0	0.263	(13)
*N*_10_(*DCP*) = *f*(*z*)	*N*_10_(*DCP*) = 12.9 + 14.2*logz* ± 2.9	0.292	(14)
*N*_10_(*DCP*) = *f*(*I_S_*)	*N*_10_(*DCP*) = −97.4 + 113.4*I_S_* ± 2.7	0.391	(15)
*PI* = *f*(*z*, *I_S_*)	*PI* = 170.9 − 16.5*z* − 159.7*I_S_* ± 4.4	0.441	(16)
*N*_10_(*DCP*) = *f*(*z*, *I_S_*)	*N*_10_(*DCP*) = 20.5 + 14.5logz + 247.4log*I_S_* ± 1.9	0.691	(17)

## Data Availability

The original contributions presented in this study are included in the article. Further inquiries can be directed to the corresponding author.
